# Supplementary material for: Biocontrol efficiency and mechanism of novel Streptomyces luomodiensis SCA4-21 against banana Fusarium wilt
Source: Microbiol Spectr. 2025 Dec 10;14(1):e02968-24. doi: 10.1128/spectrum.02968-24 (PMC12772315; doi:10.1128/spectrum.02968-24)
Supplement: Supplemental material — Fig. S1; Tables S1 to S5. [file spectrum.02968-24-s0001.docx]

Apendix A: Supplementary data

**Biocontrol efficiency and mechanism of novel *Streptomyces luomodiensis* SCA4-21 against banana *Fusarium* wilt**

**Qiao Liu^#^, Liangping Zou^#^, Yufeng Chen, Junting Feng, Yongzan Wei， Miaoyi Zhang, Kai Li, Yankun Zhao, Dengbo Zhou, Wei Wang^*^, Dengfeng Qi^*^, Jianghui Xie^*^**

Institute of Tropical Bioscience and Biotechnology, National Key Laboratory of Biological Breeding of Tropical Crops, Chinese Academy of Tropical Agricultural Sciences, Haikou, 571101, China

**Correspondence:** Dr. Wei Wang ([wangweisys@ahau.edu.cn](mailto:wangweisys@ahau.edu.cn)), Dengfeng Qi ([qidengfeng@itbb.org.cn](mailto:qidengfeng@itbb.org.cn)); Dr. Jianghui Xie ([xiejianghui@itbb.org.cn](mailto:xiejianghui@itbb.org.cn))

**Tables**

Table S1 Compounds identified from *Streptomyces luomodiensis* SCA 4-21 by GC-MS

| **No.** | **Compound Name** | RT（min） | **Probability (%)** | **Area (%)** | **CAS** | **Formula** | **Activity** | **References** |
| --- | --- | --- | --- | --- | --- | --- | --- | --- |
| 1 | (2R,3R,4S)-2,3,4-TrimethyloMethyl butanoateane | 1.347 | 34.6 | 0.896 | 32347-12-9 | C6H12O | - |  |
| 2 | Methyl butanoate | 2.333 | 89.5 | 0.5462 | 623-42-7 | C5H10O2 | Anticancer; Fruit flavor | Khan et al., 2016; Zhao et al., 2022 |
| 3 | Fluoroethene | 3.427 | 67.2 | 0.3871 | 75-02-5 | C2H3F | No activity reported |  |
| 4 | Methyl 3-methylbutanoate | 3.562 | 89.5 | 4.0036 | 556-24-1 | C6H12O2 | Nematicidal activity | Ayaz et al., 2021 |
| 5 | Methyl (E)-2-methylbut-2-enoate | 6.439 | 36.8 | 0.335 | 6622-76-0 | C6H10O2 | - |  |
| 6 | Methyl 3-methylpentanoate | 7.178 | 95 | 0.3081 | 2177-78-8 | C7H14O2 | No activity reported |  |
| 7 | Methyl 4-methylpentanoate | 7.484 | 74.6 | 1.6779 | 2412-80-8 | C7H14O2 | No activity reported |  |
| 8 | Methyl hexanoate | 8.897 | 87.2 | 1.7194 | 106-70-7 | C7H14O2 | Fruit flavor | Zhao et al., 2022 |
| 9 | 3,3-dimethylpropanethioic S-acid | 9.514 | 36 | 0.5462 | 55561-02-9 | C5H10OS | - |  |
| 11 | 5-Methyl-2-heptanone | 10.649 | 72.4 | 0.5866 | 18217-12-4 | C8H16O | Antifungal activity | Morita et al., 2019 |
| 10 | 6-Methyl-2-heptanone | 10.225 | 92.6 | 0.2866 | 928-68-7 | C8H16O | Antifungal activity | Zhang et al., 2022 |
| 12 | Methyl (2E)-2-hexenoate | 10.752 | 85.7 | 2.7822 | 13894-63-8 | C7H12O2 | Fruit flavor | Liu et al., 2018 |
| 13 | 1-(furan-2-yl)-2-hydroxyethanone | 11.049 | 45.7 | 0.5104 | 17678-19-2 | C6H6O3 | - |  |
| 14 | 3-Octanone | 11.711 | 74.1 | 1.4132 | 106-68-3 | C8H16O | Promoting growth | Dotson et al., 2020 |
| 15 | Methyl 5-methylhexanoate | 11.851 | 90.4 | 3.0927 | 2177-83-5 | C8H16O2 | No activity reported |  |
| 16 | Methyl heptanoate | 12.31 | 26.5 | 18.6055 | 106-73-0 | C8H16O2 | - |  |
| 17 | Hexanoic acid, 2-ethyl-, methyl ester（Methyl 2-ethylhexanoate） | 14.354 | 95.7 | 3.6489 | 816-19-3 | C9H18O2 | Antifungal activity | Li et al., 2023 |
| 18 | Tridecane | 15.115 | 6.7 | 3.0105 | 629-50-5 | C13H28 | - |  |
| 20 | Benzoic acid, methyl ester (methyl benzoate) | 16.83 | 75.4 | 5.0311 | 93-58-3 | C8H8O2 | Antifungal activity | Lima et al., 2018 |
| 21 | Methyl (3Z)-3-octenoate | 17.906 | 12.4 | 0.985 | 69668-85-5 | C9H16O2 | - |  |
| 22 | Methyl (2E)-2-octenoate | 20.558 | 81.3 | 2.9015 | 7367-81-9 | C9H16O2 | No activity reported |  |
| 23 | 2-Methylisoborneol | 21.309 | 85.2 | 0.4854 | 2371-42-8 | C11H20O | Earthy and musty taste | Abd et al., 2022 |
| 24 | 3-Phenyl-2-butanone | 23.745 | 46.9 | 0.7358 | 769-59-5 | C10H12O | - |  |
| 25 | 6-Ethyl-2-methyldecane | 25.199 | 10.6 | 0.4875 | 62108-21-8 | C13H28 | - |  |
| 26 | 1-Chlorohexadecane | 26.464 | 13.2 | 1.592 | 4860/3/1 | C16H33Cl | - |  |
| 27 | 2-N'-pyridin-2-ylethanedihydrazide | 26.743 | 6.6 | 0.3227 | 329788-02-5 | C7H9N5O2 | - |  |
| 28 | Dodecyl hexyl sulfite | 27.284 | 9 | 0.9704 |  | C18H38O3S | - |  |
| 29 | 3-(2,4,6-Cycloheptatrien-1-yl)-2,4-pentanedione | 28.441 | 59.8 | 2.0094 | 65548-56-3 | C12H14O2 | No activity reported |  |
| 30 | 4-Ethenyl-1,4-dimethyl-3-(2-methylprop-1-enyl)cycloheptene | 28.688 | 5.6 | 0.9217 |  | C15H24 | - |  |
| 31 | 5-Hexyldihydro-2(3H)-furanone | 31.759 | 35.6 | 1.5371 | 706-14-9 | C10H18O2 | - |  |
| 32 | (1aR,4R,4aR,7R,7aS,7bS)-1,1,4,7-tetramethyl-2,3,4a,5,6,7,7a,7b-octahydro-1aH-cyclopropa[e]azulen-4-ol | 36.049 | 30.4 | 0.909 | 489-41-8 | C15H26O | - |  |

Table S2 Evaluation of bacterial richness and diversity for nine banana rhizosphere soil samples from three treatments

| **Sample ID** | **OTU** | **Shannon** | **Simpson** | **Chao1** | **ACE** |
| --- | --- | --- | --- | --- | --- |
| SLM | 1732 | 9.51 | 1 | 1732 | 1734 |
| SLM | 1899 | 9.77 | 1 | 1899 | 1902 |
| SLM | 1862 | 9.64 | 1 | 1862 | 1865 |
| Foc TR4+SLM | 1946 | 9.48 | 0.99 | 1946 | 1946 |
| Foc TR4+SLM | 1863 | 9.67 | 1 | 1863 | 1866 |
| Foc TR4+SLM | 1851 | 9.48 | 0.99 | 1851 | 1854 |
| Foc TR4+SCA4-21 | 1602 | 9.19 | 0.99 | 1602 | 1604 |
| Foc TR4+SCA4-21 | 1852 | 9.3 | 0.99 | 1852 | 1853 |
| Foc TR4+SCA4-21 | 1647 | 9.2 | 0.99 | 1647 | 1649 |

Table S3 Relative abundance of bacterial genera of different treated soil sample

| **genus** | **SLM (%)** | **Foc TR4+SLM (%)** | **Foc TR4+SCA4-21(%)** |
| --- | --- | --- | --- |
| unclassified-*Xanthobacteraceae* | 5.32 | 4.97 | 2.29 |
| unclassified-*Acidobacteriales* | 5.49 | 3.54 | 2.19 |
| *Bacillus* | 0.3 | 0 | 9.55 |
| unclassified-*Bacteria* | 3.17 | 3.37 | 1.78 |
| unclassified-*Cyanobacteriales* | 1.06 | 5.16 | 0.32 |
| *Burkholderia-Caballeronia-Paraburkholderia* | 1.81 | 0.9 | 3.75 |
| unclassified-*Elsterales* | 3.37 | 2.18 | 0.85 |
| *Haliangium* | 2.53 | 2.43 | 1.34 |
| *Pantoea* | 1.09 | 4.74 | 0.05 |
| *Bryobacter* | 3.22 | 1.38 | 0.96 |
| unclassified-*Gemmatimonadaceae* | 1.94 | 2.3 | 1.31 |
| *Sphingomonas* | 0.91 | 0.82 | 2.95 |
| *Cupriavidus* | 0.62 | 0.48 | 3.37 |
| unclassified-*Comamonadaceae* | 1.18 | 2.12 | 1.14 |
| unclassified-SBR1031 | 2.49 | 1.52 | 0.25 |
| unclassified-*Alphaproteobacteria* | 1.86 | 1.73 | 0.63 |
| *Massilia* | 0.18 | 0.05 | 3.98 |
| *Pseudomonas* | 0.33 | 0.12 | 3.71 |
| uncultured-*Acidobacteria-bacterium* | 1.58 | 1.74 | 0.59 |
| *Streptomyces* | 0.48 | 0.31 | 3 |
| Others | 61.05 | 60.1 | 55.96 |
| Unknown | 0.01 | 0.01 | 0.02 |

Table S4 Evaluation of fungal richness and diversity for nine banana rhizosphere soil samples from three treatments

| **Sample ID** | **OTU** | **ACE** | **Chao1** | **Simpson** | **Shannon** |
| --- | --- | --- | --- | --- | --- |
| SLM | 309 | 309 | 309 | 0.9 | 4.59 |
| SLM | 411 | 411 | 411 | 0.97 | 5.97 |
| SLM | 591 | 591 | 591 | 0.95 | 5.93 |
| Foc TR4+SLM | 316 | 316 | 316 | 0.81 | 4.35 |
| Foc TR4+SLM | 446 | 446 | 446 | 0.85 | 4.65 |
| Foc TR4+SLM | 429 | 429 | 429 | 0.85 | 4.93 |
| Foc TR4+SCA4-21 | 413 | 413 | 413 | 0.88 | 5.29 |
| Foc TR4+SCA4-21 | 341 | 341 | 341 | 0.99 | 7.47 |
| Foc TR4+SCA4-21 | 480 | 480 | 480 | 0.95 | 6.08 |

Table S5 Relative abundance of fungal genera of different treated soil sample

| **genus** | **SLM (%)** | **Foc TR4+SLM (%)** | **Foc TR4+SCA4-21(%)** |
| --- | --- | --- | --- |
| Fusarium | 6.65 | 37.29 | 16.92 |
| unclassified-*Sordariomycetes* | 7.34 | 21.36 | 6.98 |
| unclassified-*Basidiomycota* | 15.13 | 3.68 | 12.06 |
| unclassified-*Ascomycota* | 7.56 | 6.78 | 3.89 |
| unclassified-*Agaricomycetes* | 8.96 | 6.35 | 1.58 |
| unclassified-*Fungi* | 4.59 | 3.35 | 3.77 |
| unclassified-*Dothideomycetes* | 1.52 | 4.03 | 1.17 |
| *Gymnopilus* | 5.56 | 0.01 | 0 |
| *Conocybe* | 2.43 | 0.65 | 2.48 |
| *Gibellulopsis* | 0.39 | 0.3 | 2.51 |
| *Xenomyrothecium* | 0.12 | 0.16 | 2.74 |
| unclassified-*Thelephoraceae* | 2.67 | 0 | 0 |
| *Purpureocillium* | 0.34 | 0 | 2.28 |
| *Lycoperdon* | 1.3 | 0 | 1.28 |
| *Mortierella* | 0.23 | 0.06 | 2 |
| *Cladosporium* | 0.52 | 0.33 | 1.41 |
| unclassified-*Glomeraceae* | 0.86 | 0.68 | 0.33 |
| *Rhizophagus* | 1.11 | 0.54 | 0.16 |
| *Arthrobotrys* | 0.05 | 0.17 | 1.27 |
| unclassified-*Glomeromycota* | 0.56 | 0.54 | 0.34 |
| Others | 5.22 | 5.47 | 28.38 |
| unidentified | 26.89 | 8.24 | 8.46 |

Figures


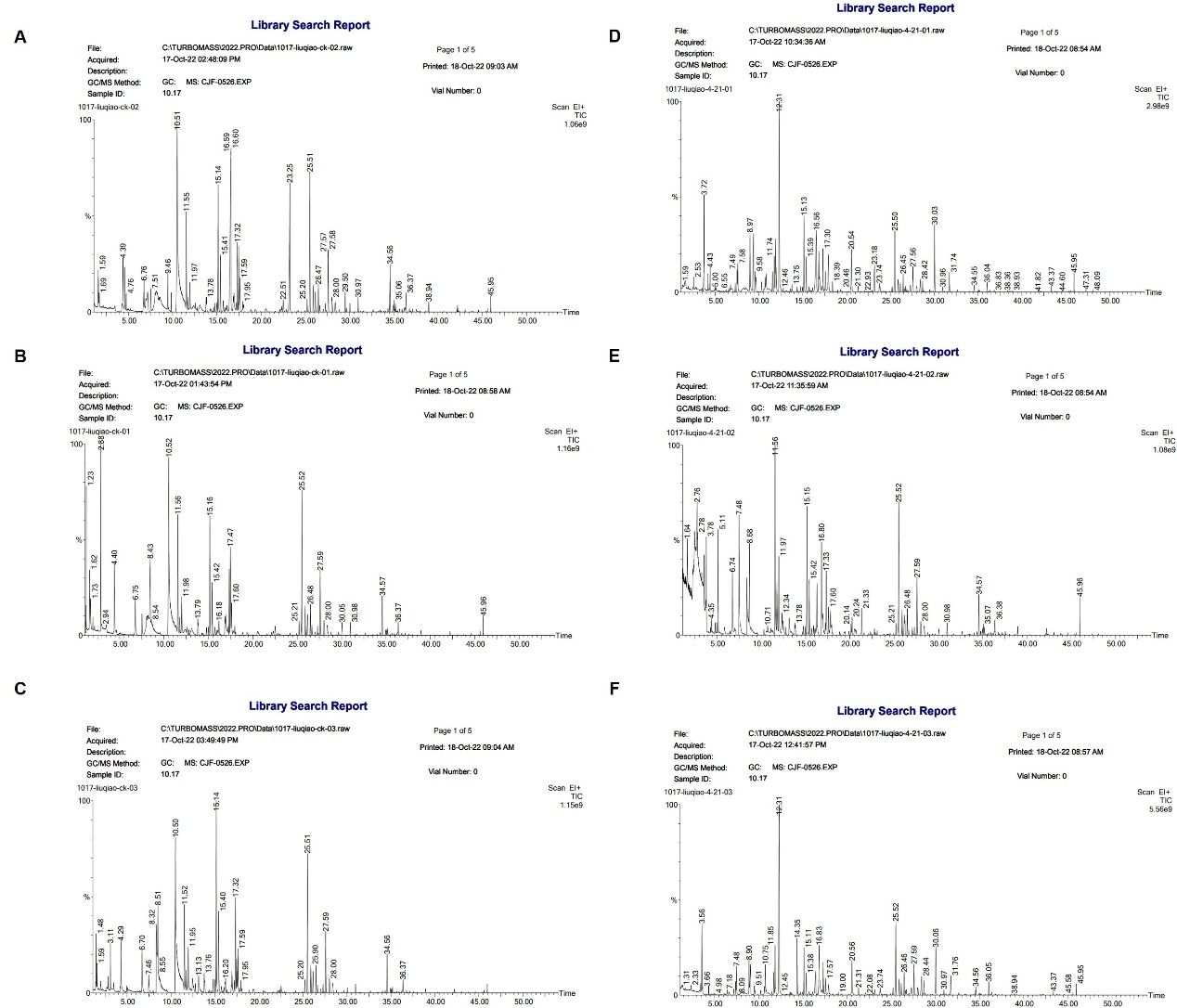


Fig. S1. Analysis of gas chromatography-mass spectrometry volatile organic compounds in *S. luomodiensis* SCA4-21. (A, B, C) GC-MS chromatogram for controls (sterilized soybean liquid medium). (D, E, F) GC-MS chromatogram for treatments (fermentation broth of *luomodiensis* SCA4-21).

**References**

Zhao, H., Ren, L., Shen, R., Guo, S., Peng, X., 2022. Identification of the influential odorants for the unpleasant rancid smell of ripe noni fruit (*Morinda citrifolia*). Int. J. Food Sci. Tech. 57(4), 2277-2284. <https://doi.org/10.1111/ijfs.15578>

Liu, Y., Dong, W., Zhang, F., Kenis, M., Griepink, F., Zhang, J., Chen, L., Xiao, C., 2018 Identification of active components from volatiles of Chinese bayberry, *Myrica rubra* attractive to *Drosophila suzukii*. Arthropod-Plant Inte. 12, 435-42. https://link.springer.com/article/10.1007/s11829-018-9595-z

Oh, H. S., Lee, C. S., Srivastava, A., Oh, H. M., Ahn, C. Y., 2017. Effects of environmental factors on cyanobacterial production of odorous compounds: geosmin and 2-methylisoborneol. J. Microbiol. Biotechnol. 27(7), 1316-1323. https://doi.org/10.4014/jmb.1702.02069
